# Sustaining BEST in CLASS: Teacher-Reported Evidence-Based Practice Use with Students at Risk for Emotional and Behavioral Disorders Amidst the COVID-19 Pandemic

**DOI:** 10.1007/s12310-022-09561-y

**Published:** 2022-12-20

**Authors:** Melissa Washington-Nortey, Kristen Granger, Kevin S. Sutherland, Maureen Conroy, Navneet Kaur, Allyse Hetrick

**Affiliations:** 1 Department of Psychology, Institute of Psychiatry, Psychology, and Neuroscience, King’s College London, Addison House, Guy’s Campus, London, SE1 1UL UK; 2 Department of Special Education, Peabody College, Vanderbilt University, 230 Appleton Place, Nashville, TN 37203 USA; 3 Department of Counseling and Special Education, School of Education, Virginia Commonwealth University, Box 842020, Richmond, VA 23284-2020 USA; 4 Anita Zucker Center for Excellence in Early Childhood Studies, University of Florida, PO Box 117050, Gainesville, FL 32611 USA; 5 Department of Psychology, Virginia Commonwealth University, Box 842020, Richmond, VA 23284-2020 USA; 6 Institute for Child Health Policy, University of Florida, 2004 Mowry Rd, Gainesville, FL 32603 USA

**Keywords:** Evidence-based practices, Emotional or behavioral disorders, Sustainment, Elementary students

## Abstract

There is growing evidence of the efficacy of evidence-based interventions in improving the academic and social outcomes of children who exhibit challenging behaviors during program implementation periods. However, less is known about the extent to which practices learned as part of these interventions are sustained after these projects end, when funding is paused temporarily, and in less-than-ideal conditions. This study used qualitative methods to investigate whether teachers previously trained in the BEST in CLASS-Elementary intervention continued to use the program’s evidence-based practices with students 1–2 years after completing the program and in the context of the COVID-19 pandemic. It also examined teachers’ perceptions of the impact of practice use on students’ academic and social outcomes. Thirteen BEST in CLASS-Elementary teachers from elementary schools in two southeastern states in the USA where the program was implemented completed semi-structured interviews on the topic. Data were coded thematically, and the results indicated that over 50% of teachers reported using “rules,” “supportive relationships,” and “praise” frequently with their students. However, “precorrection” and “opportunities to respond” were reportedly used less often. Teachers also perceived that their use of these evidence-based practices was linked to increases in their students’ academic engagement and academic performance and knowledge, improvements in students’ behaviors, their relationships with teachers, and general comfort and self-confidence. The discussion highlights modality-specific patterns noted in the results that might influence sustainment and the implication of these findings for interventions and programs aimed at promoting positive behavioral outcomes for early elementary school students.

## Introduction

Many students enter elementary school experiencing moderate to severe problem behaviors and have difficulty adjusting to their educational environment (Ringeisen et al., 2020). Almost 10% of elementary-aged students experiencing these difficulties will require support to overcome these problem behaviors (Forness et al., 2012). These rates provide documentation that students with problem behaviors are most likely present in most early elementary classrooms in the USA (Costello et al., 2005, 2006; Forness et al., 2012), and these behavioral challenges impact students’ future adjustment and school success. To illustrate, early onset of behavior problems in students predict later drug abuse, juvenile delinquency, violence, school dropout, peer rejection, and poor academic outcomes, among other deleterious outcomes (Beyer et al., 2012; Campbell et al., 2006; McClelland et al., 2007; Shonkoff & Phillips, 2000). The intensity and severity of problem behaviors increase over time, making the early elementary grades a critical and sensitive period for intervention (Beyer et al., 2012; Dunlap et al., 2006; Marchant et al., 2004).

Positive Behavioral Interventions and Supports (PBIS) is a three-tiered framework that guides the provision of evidence-based intervention supports to improve students’ behavioral and academic outcomes (Kowalewicz & Coffee, 2014; Sugai & Horner, 2009; Sugai & Simonsen, 2012; Williford et al., 2015). In elementary settings, the efficacy of evidence-based Tier 2 intervention programs—interventions targeting students at risk to prevent and mitigate young students’ chronic problem behaviors—have shown that evidence-based interventions, which include specific intervention practices, can be used within teacher–student interactions to address students’ problem behaviors and improve their social-emotional learning skills (e.g., Campbell et al., 2013). However, less is known about the extent to which teachers continue to use these practices after program professional development supports are removed due to an expected end in program implementation or challenging circumstances that result in unplanned definite or indefinite pauses to implementation. For instance, in 2020, measures to mitigate the spread of COVID-19 affected educational settings; compelling many schools to transition to online learning environments. These measures also confronted teachers with increased psychological demands, greater workloads, and limited resources, contributing negatively to their mental health and well-being (Kim & Asbury, 2020). Of note, some in-person school-based interventions were also forced to pause temporarily as they were not designed for virtual implementation. These stressors provided a unique opportunity to learn about the extent to which teachers use evidence-based practices with students expressing challenging behaviors under highly stressful teaching conditions with limited support.

The present study focuses on teachers who participated in a Tier 2 program, BEST in CLASS-Elementary, which includes a practice-based coaching professional development component designed to support elementary school teachers’ use and delivery of evidence-based practices with students at risk for emotional and behavioral disorders (EBD; Sutherland et al., 2020a). Specifically, we examine teacher reports of sustained use and effects of the BEST in CLASS practices one-to-two years after teachers completed the BEST in CLASS intervention, including the practice-based coaching component. Following teachers after their participation in the BEST in CLASS intervention is meaningful as it allows for an examination of treatment effects after research support, including coaching, has been withdrawn. Importantly, we assess these questions within the context of the COVID-19 pandemic.

### Sustained Use of Evidence-Based Practices

Research examining sustained use and the sustained impact of Tier 2 classroom-based programs has produced mixed results. To illustrate, Bierman et al. (2013) examined the extent to which high-quality teaching practices and the use of an evidence-based intervention were maintained a year after completing the Research-based Developmentally Informed (REDI) project among preschool teachers. Thirty-seven teachers participated in a mixed-method study, including quantitative and qualitative follow-up assessments probing sustained use and effects. The results showed that sustainment patterns differed by intervention components. Specifically, whereas the use of the Promoting Alternative Thinking Curriculum (PATHS; Domitrovich et al., 2005) intervention component was sustained with high-quality, there were declines in teachers’ use of the literacy/language intervention components. Further, teachers’ overall quality of instruction at baseline predicted sustainment, and teachers who demonstrated higher levels of sustained implementation in one domain were likely to maintain practice use in other domains (Bierman et al., 2013).

When examining the effects of sustained intervention practice use, some studies report long-term effects on student outcomes while others do not. For instance, Diken and Rutherford (2005) found that at 2 months post-intervention, participation in First Step to Success (Walker et al., 1997) was associated with increases in students’ positive social behaviors and decreases in nonsocial behaviors. On the other hand, Overton et al. (2002) obtained mixed results when they examined the sustained effect of the same intervention, implemented with a different sample, at 1-year post-intervention; some students continued to show decreases in externalizing behaviors, while others showed increases. Woodbridge et al. (2014) similarly documented that the initial positive behavioral effects of First Step to Success intervention among early elementary students were not sustained. Instead, compared to students in the control group, intervention group students evidenced significantly poorer outcomes on five of the six behavioral measures assessed and in the academic domain. This research illustrates that effects may be difficult to sustain, and, in some cases, child outcomes may even have returned to baseline levels (e.g., Altman, 2009). To prevent a decrease or reversal of effects, current intervention research efforts emphasize engaging in strategies that not only strengthen intervention implementation but also increase the likelihood of sustainment after implementation (Hailemariam et al., 2019).

Knowledge about the implementation and sustainment of intervention programs is growing. For example, Domitrovich et al. (2008) proposed a multi-level framework of factors that may influence the quality of program implementation in schools. This model is consistent with a socio-ecological framework (Bronfenbrenner, 1977) and takes into consideration the influences of macro-level (e.g., government policies, community supports, funding), school-level (e.g., school policies, school climate, and school resources), and individual-level factors (e.g., teacher self-efficacy, level of education, intervention acceptability). The factors at each level are interdependent with the potential to influence the quality with which interventions are implemented, although proximal factors are thought to be more influential than distal factors. For example, school-based interventions are more likely to be sustained when they meet specific school-related criteria; when interventions meet expressed needs of school staff; are perceived as being beneficial to the students; fit the school context, and as such, are easily integrated into the school’s regular protocol and systems; and when the intervention protocol is perceived as practical and feasible (Forman et al., 2009; Han & Weiss, 2005). Further, interventions implemented over a long period are more likely to be sustained (McIntosh et al., 2015). Although knowledge about sustainment is growing, few interventionists have examined whether their program’s effects are sustained after implementation. Therefore, this study sought to examine the sustained effect of the BEST in CLASS-Elementary intervention. In the next section, we highlight the implementation of BEST in CLASS to contextualize the present study.

### BEST in CLASS

BEST in CLASS is a Tier 2 teacher-delivered intervention that promotes students’ social-emotional and behavioral competence through the development of positive teacher–child interactions. BEST in CLASS promotes teachers’ increased use of effective instructional practices through professional development, including individualized practice-based coaching, which promotes the key mechanisms of change: positive teacher–child interactions and improved teacher–child relationships. In elementary school classrooms, the relationship between a teacher and student is an important factor in the learning environment that contributes to student success, fostering positive academic and social-emotional competencies (Pianta et al., 2012). High-quality relationships with teachers may be particularly important for students with or at risk of EBD. Students with or at risk for EBD can often develop coercive interactions with their teachers, which may contribute to cycles of problem behavior and continued conflict (Sutherland & Oswald, 2005). To illustrate, teacher–student interactions remained negative with students exhibiting high rates of disruptive behavior a year after externalizing behaviors were identified (Henricsson & Rydell, 2004). These students may have more to gain from high-quality positive interactions and relationships with teachers compared to their peers (Belsky, 1997; McGrath & Bergen, 2015). BEST in CLASS-Elementary is designed to support positive teacher–student interactions by increasing teachers’ frequency and quality of use of evidence-based practices shown to decrease student problem behavior and increase engagement. These classroom-based practices include (a) Supportive Relationships, (b) Rules, (c) Precorrection, (d) Opportunities to Respond, and (e) Praise. Supportive relationships provide teachers with strategies to positively respond to students who are having difficulty regulating their emotions, which strengthen their positive relationships with the students. Rules are guidelines that communicate behavioral expectations and provide focal students with a structure for engaging in activities throughout the school day. Precorrection is a preventive practice that addresses anticipated challenging behaviors before they occur by reminding focal students of the expected appropriate behaviors before an activity starts. Opportunities to respond are questions or requests made to the focal students that warrant clear, observable responses and increase their engagement in activities. Lastly, praise informs students about their specific behaviors that are expected and typically results in an increase in the likelihood of those behaviors occurring in the future. Although these practices are typically used by teachers, BEST in CLASS practice-based coaching supports teachers to use these practices intentionally, at a higher frequency, and with greater quality with focal students in the classrooms (compared to their classmates) to increase the likelihood of appropriate behaviors (Conroy et al., 2019).

BEST in CLASS has been found to be effective during implementation years in both early childhood and elementary school settings (Sutherland et al., 2018, 2020a, 2020b). To illustrate, BEST in CLASS has been shown to be effective at improving preschool-age child (Conroy et al., 2015; Sutherland et al., 2018) and early childhood teacher outcomes (Conroy et al., 2019). Findings from a randomized controlled trial conducted with 186 early childhood teachers revealed reductions in teacher-reported problem behaviors, improved teacher–child closeness, and reduction in teacher–child conflicts compared to the Business as Usual (BAU) group (Sutherland et al., 2018). Additionally, teachers reported an increase in self-efficacy and overall classroom quality (Conroy et al., 2019). Similarly, findings from a randomized controlled trial with 26 teachers in elementary school classrooms revealed decreases in problem behaviors reported by teachers and improvements in teacher–student closeness compared to the BAU group (Sutherland et al., 2018). Additionally, teachers in BEST in CLASS-Elementary condition reported less emotional exhaustion compared to the control condition (McCullough et al., 2021). However, the extent to which teachers continue using the BEST in CLASS evidence-based practices after the intervention is complete and its perceived impact on child outcomes is unknown.

Moreover, in challenging educational contexts/circumstances, as experienced during the COVID-19 pandemic, teachers may adopt new strategies or modify previously used strategies. For example, during the COVID-19 pandemic, many schools quickly changed their instructional format to online learning. For many teachers, the transition was stressful as the uncertainty of the pandemic meant that many schools only decided on an instructional modality close to the beginning of the academic year, limiting teachers’ planning time. Limited familiarity with technology, the need to share workspaces with family members and, in some cases, attend to their own children’s needs while teaching, also affected teachers’ stress levels during the period (MacIntyre et al., 2020). Further, the BEST in CLASS practices were not tested or used in an online format during the implementation year; as such, it remains an open question if teachers, who shifted to providing online instruction, used these evidence-based practices. Other teachers who maintained in-person instruction also faced several novel challenges such as adhering to new regulations and mandates that may have impacted their willingness and ability to implement evidence-base practices. Given the added pressures teachers faced during the period and the removal of external supports, teachers might have reverted to previous strategies in place of the BEST in CLASS practices. Also, little is known about teachers’ perceptions of the impact of these practices under such stressful conditions. An assessment of the extent to which teachers continue to use these practices during difficult circumstances provides an opportunity to assess BEST in CLASS sustainment and potential benefits in less optimal conditions.

### Present Study

The purpose of this study is to assess the extent to which teachers deliver BEST in CLASS practices that are designed to support elementary school students with or at risk for EBD one-to-two years after intervention participation. Additionally, we examined teacher reports on the extent to which they perceived these practices to influence student performance and behavior. Specifically, we focus on two research questions:

1. To what extent do teachers who participated in BEST in CLASS report using the BEST in CLASS practices one-to-two years later (in the 2020–2021 school year)?
2. What are teachers’ perspectives of the extent to which BEST in CLASS practices are associated with students’ academic performance, behaviors, and social skills?

Importantly, we took a qualitative approach to these research questions because the limited evidence on sustainment patterns in Tier 2 interventions and their effect on child outcomes hindered our ability to test a clear hypothesis. Qualitative inquiries are more suited to exploratory investigations where data is limited (Braun & Clarke, 2013). Using a qualitative approach also enabled us to assess teachers' perceptions of impact across more broadly defined constructs. Lastly, adopting a qualitative approach equipped the team with foundational evidence that can inform further sustainment inquiries. Qualitative inquiries are often used to obtain such foundational evidence to inform larger-scale quantitative inquiries (Braun & Clarke, 2013).

## Method

### Design

We used descriptive phenomenology via semi-structured interviews to explore teachers’ use of the BEST in CLASS practices and their perspectives of its impact on themselves and a focal student in their classroom whom they perceived to be at risk for or have EBD. Interviews were conducted by two female postdoctoral researchers and one female research faculty member who were all associated with the project. On average, the interviews, which were held and recorded on ZOOM (Zoom Video Communications, 2020), lasted between one hour to one hour 30 min each.

### Setting, Participants, and Sampling

Schools in two southeastern states in the USA that had previously participated in the BEST in CLASS intervention program served as the sampling frame for this study. At the time of data collection, BEST in CLASS had been implemented in four schools in state A and three schools in state B. All schools in state A were located in a single urban school district. On average, enrollment rates ranged between 162 and 576 students (average 326 students) at each school, 72% (range 66–99%) of which were eligible for free or reduced lunch from the federal government based on federal guidelines. Approximately 80% of students (range 46–95%) were from Black or African American racial/ethnic backgrounds. All schools in state A used virtual instruction during the 2020–2021 academic year. On the other hand, all schools in state B were located in a rural district and served 643 (range 561–766) students on average. Approximately 78% (range 63–100%) of students were eligible for free or reduced lunches and most students in these schools were White (i.e., average 80% white, 9% Black, and 8% Hispanic/Latino). Intervention schools in state B taught classes using a variety of formats to accommodate students. Whereas most students (~ 80%) were taught in-person, the remainder opted for hybrid instruction or a purely online format during the 2020–2021 academic year.

Teachers who had participated in the BEST in CLASS condition in a multi-site, randomized controlled trial in the previous two academic years (*n* = 31) were recruited using email correspondence and referrals from other BEST in CLASS participants and BEST in CLASS project staff. They were then sampled using convenience sampling approaches. Thirteen previous BEST in CLASS teachers, all licensed to teach, consented to participate in this study. These included all teachers who responded to email invitations and teachers who were reminded by their colleagues or BEST in CLASS staff about the emailed invitations. All teachers who responded to our call and expressed an interest in the study were scheduled for an interview at a time convenient to them. A larger number of teachers who had participated in the intervention during the 2019–2020 academic year expressed interest in the qualitative study (*n* = 11) than teachers who had completed the intervention in the 2018–2019 academic year (*n* = 2). Each teacher participated in the intervention for a single academic year. See Table 1 for information on teachers’ demographic characteristics.

**Table 1:** Teachers’ demographic characteristics

	Intervention—Y1 teachers (*n* = 2)	Intervention—Y2 teachers (*n* = 11)
Gender		
Female	2	11
Male	0	0
Race/ethnicity		
African American/Black	0	2
Caucasian/White	2	9
Hispanic/Latino	0	0
Age range		
26–35	2	4
36–45	0	5
46–55	0	2
Over 55	0	0
Grade level assignment		
K	1	2
1	0	4
2	0	0
3	0	3
Other	1	2
Highest level of education		
Bachelor’s	0	7
Master’s	2	3
Other	0	1

	*M* (SD)	*M* (SD)
Years teaching current grade	.50 (.71)	4.18 (4.23)
Years teaching total	3.5 (.71)	14.27 (6.47)

Before engaging in the interviews, teachers completed consent procedures online with REDCap (Harris et al., 2009). The study procedures and interview protocol were approved by each university’s Institutional Review Board. Before each interview, teachers were requested to identify a current student in their classroom who exhibited challenging behaviors similar to students found eligible for participation during the BEST in CLASS implementation year. Teachers were subsequently requested to answer all interview questions in reference to the specific student identified. For this manuscript, we analyzed questions assessing teachers’ perspectives on their use of BEST in CLASS practices and of its impact on their students’ academic performance, behaviors, and social skills. The specific questions are listed in Table 2.

**Table 2:** Specific research questions analyzed in this study

Interview questions	
1.	When you think of (student name), which BEST in CLASS evidence-based practices have you used most frequently this year?
2.	How has your use of BEST-in-CLASS evidence-based practices impacted (student name) in regard to behaviors and social skills?
3.	How has your use of BEST-in-CLASS evidence-based practices impacted (student name) in regard to academic performance?

### Data Analytic Procedure

Interviews were audio and video recorded, transcribed verbatim through a transcription service, and verified by members of the coding team before data were coded. Data were coded by a nine-member team that included doctoral-level researchers (*n* = 2), advanced graduate (*n* = 2) and undergraduate (*n* = 3) students, and two coders with bachelor’s degrees. There were seven females and two males from African (*n* = 1), African American (*n* = 3), White (*n* = 2) Spanish (*n* = 1), and Chinese (*n* = 2) backgrounds on the coding team. A comprehensive training occurred for coders that involved reading seminal articles on qualitative analysis, participating in virtual training sessions, and receiving direct supervision from doctoral-level researchers with extensive qualitative experience. First, coders read Braun and Clarke’s (2006) article on thematic coding analysis and Patton’s (2005) article on qualitative research before meeting together for the virtual training sessions. There were three parts to the virtual training session; in the first part, coders reflected on their readings, listened to a presentation on the general principles of qualitative research, watched video presentations on open and axial coding strategies, and practiced these strategies with their team members using examples from previous studies. The second and third parts of the training related directly to the software used in coding, ATLAS ti (Version 8.4.26). Part two introduced coders to the software and its functionality. This was deemed important as many coders were new to qualitative coding and qualitative coding software. In part three, coders received specific instruction on how to use the software to code and keep a memo of the reflections after each coding session. In the final training component, coders held independent meetings with the supervisor after their first training exercise to review each code and obtain feedback on strategies to improve their coding skills. The supervisor was also present at all initial consensus coding meetings to observe the process and provide feedback where necessary. Lastly, coders consulted with the supervisor throughout the coding process to discuss questions and concerns, and team members met weekly with the supervisor to debrief and discuss emergent patterns they had noticed while coding.

### Coding Process

We used Braun and Clarke’s (2006) six-step data-driven thematic analysis strategy to code the data. First, coders worked independently on specific coding assignments. Next, two coders, assigned to the same task, worked in pairs to code the data by consensus. Finally, after all consensus coding assignments were complete, the entire team met to assess and review the text-code pairs and the emergent themes that had been extracted. Coding comprised the following steps: (1) familiarizing ourselves with the data, (2) generating initial codes, (3) searching for themes, (4) reviewing the themes, (5) defining and refining the names of the themes, and (6) producing the report. Coders independently familiarized themselves with the data by verifying the transcribed data and reading the full transcript of each interview before initiating coding. They also worked independently, using open coding strategies (Strauss & Corbin, 1994) to generate initial codes. This involved identifying relevant, codable units of text in the data and labeling these extracted texts with preliminary essence-capturing codes. Next, they met with their paired team members, assigned to the same task, to complete consensus open coding by discussing all text-code pairs and developing a finalized list of consensus coded texts.

Searching for themes was completed using axial coding strategies. At this stage, the supervisor carefully examined the coded texts to identify patterns, relationships, and emergent themes and sorted all the initial codes into different themes and subthemes based on their conceptual similarities and differences. These results were presented to the coding team for review and feedback. The team collectively reviewed the themes and subthemes by examining all the coded texts under each theme and subtheme for coherence. The team refined the themes, subthemes, and their associated names based on feedback generated during this process. Negative cases, one-off texts that did not align with the final theme structure, were also agreed upon and eliminated by team consensus. Based on the discussions, team members also drafted and revised the definitions of each theme and subtheme. Definitions and frequency counts evidencing theme and subtheme representation in the data are presented in Table 3. We deviated from this approach slightly to answer research question 1, as it did not require theme generation. Instead, we used open and consensus coding strategies to conduct a content analysis and obtain a tally of the frequency with which teachers reported using BEST in CLASS practices with their focal students.

**Table 3:** Impact of BEST in CLASS practices on students’ behaviors, social skills, and academic performance

Themes and subthemes	Number of BIC participants	Number of State A participants	Number of State B participants
	Teachers (*n* = 13)	Teachers (*n* = 4)	Teachers (*n* = 9)
Theme 1: Increased academic engagement—Refers to participant’s assertions that using BEST in CLASS practices increased student's willingness to participate in or attempt tasks and remain engaged and focused throughout the period. It also includes specific references to methods that teachers used to foster increased engagement	10 [77%]	1 [25%]	9 [100%]
Subtheme 1.1: Increased academic motivation and effort—Refers to student’s increased willingness and capacity to start, maintain, and complete regular and subjectively challenging academic tasks without getting frustrated	6	1	5
Subtheme 1.2: Increased attention and focus—Refers to students paying more attention to teachers' instruction, ongoing classroom activities, and focusing on tasks for longer periods than usual	3	0	3
Subtheme 1.3: Increased engagement and participation in-class activities—Refers to teachers’ efforts at increasing participating and a noted increase in students’ likelihood of joining classroom discussions, engaging in lessons/academic tasks, carrying out classroom routines, and sharing their thoughts or experiences with class	6	1	5
Theme 2: Improved academic knowledge and performance—Refers to students improving academic performance in general or in specific subjects, and students having an improved understanding and knowledge base	6 [46%]	1 [25%]	5[56%]
Subtheme 2.1: Improved academic skills and performance—Refers to more general comments about students making progress in academics, completing more tasks, and having better overall academic performance	5	1	4
Subtheme 2.3: Improved students’ understanding and knowledge base—Refers to students demonstrating an increased knowledge base and better understanding of academic content	1	1	0
Theme 3: Improved students' behavior and the quality of student–teacher relationships—Refers to the teacher providing students with behavioral expectations and students engaging in more positive behaviors	11 [84%]	3 [75%]	8 [89%]
Subtheme 3.1: Clarified expectations about appropriate behavior—Refers to teacher reminders of the behavioral expectations and classroom rules before starting an activity, before a student engages in challenging behaviors, or after a student exhibits challenging behaviors	4	0	4
Subtheme 3.2: Improved students’ general behavior—Refers to teacher-perceived improvements in student behavior based on compliance to expectations or a general positive impact on student behaviors	8	2	6
Subtheme 3.3: Helped nurture positive student–teacher relationships—Refers to teachers and students becoming more attached to and friendly with each other, as well as students becoming more comfortable engaging with their teacher on personal issues	8	3	5
Theme 4: Increased confidence and general comfort—Refers to students feeling comfortable making new friends or interacting with other students, acting more confident during interactions, and attempting challenging tasks or activities	2 [15%]	0	2 [22%]
Theme 5: No or unclear effects on academic achievement—Refers to teachers’ uncertainty about or perceived absence of effects on students' academic performance	4 [31%]	0	4 [44%]

N.B. The frequency counts associated with the main and subthemes refer to the number of participants whose comments fit within that theme out of the total number of participants interviewed. The percentages associated with the main themes refer to the proportion of participants whose comments fit within that theme out of the total number of participants interviewed

### Positionality

Qualitative research encourages researchers to reflect on the identities represented in the team and their potential impact on the results (Bourke, 2014). Our research team, including the coding team, was diverse. It comprised undergraduate and graduate students, research assistants with undergraduate degrees, early-career academic researchers, and experienced faculty members. The team was also diverse in gender representation, racial/ethnic background, and level of experience with qualitative research. These factors may have influenced our results and the discussion of these results. For instance, more experienced members of the team may have been more invested in the project and eager to identify evidence of sustained use of the practices and impact. However, we took several steps, outlined in the next section, to ensure the trustworthiness of our results.

### Trustworthiness of the Data

To ensure trustworthiness we engaged in several recommended practices (Connelly, 2016; Patton, 1999). For instance, we included multiple investigators in coding the data, some of whom were involved in the data collection process; consequently, these coders could provide further context in situations of ambiguity. The coding team met weekly to debrief and discuss emerging patterns and concerns. At these meetings, members could openly question, agree, or disagree with emerging codes or themes. Coders were also encouraged to keep active memos about their coding process which informed the debriefing sessions. Further, we used member-checking procedures to critically evaluate our themes and subthemes and refine them as needed. This helped reduce the potential impact of individual biases and increase data credibility and confirmability (Connelly, 2016).

## Results

### BEST in CLASS Practice Use

All teachers reported that they used at least one of the five BEST in CLASS practices assessed (i.e., Supportive Relationships, Rules, Precorrection, Opportunities to Respond, and Praise) with their focal student, and one teacher reported using all five practices with her student. The largest proportion of teachers (76%) reported using rules most frequently with their students. The second and third most frequently used practices were supportive relationships (used by 61% of teachers) and praise (used by 53% of teachers), respectively. Fewer teachers (39%) reported using precorrection and opportunities to respond (39%) with their focal students.

### Impact of BEST in CLASS Practice Use on Student Outcomes

The data yielded four main themes, with associated subthemes, demonstrating the perceived impact of using BEST in CLASS practices on students’ academic performance, behaviors, and social skills. Specifically, the majority of teachers shared that from their perspectives, using the BEST in CLASS practices *(1) increased student’s academic engagement, (2) improved student’s academic knowledge, skills, and performance, (3) improved student’s behavior, and (4) improved student’s comfort and general confidence in the classroom.* However, a few teachers expressed uncertainty about whether the practices positively impacted their students. These teachers’ perspectives were captured in the last theme*: unclear effects.* Definitions and frequency reports showing their general and site-specific representation in the data are presented in Table 3.

The first theme, *increased academic engagement*, comprised three subthemes including, *(1) increased academic motivation and effort, (2) increased attention and focus,* and *(3) increased engagement and participation in-class activities.* Over 75% of teachers across both sites endorsed this theme. Within sites, 25% of teachers in state A and all the teachers in state B indicated that they perceived their use of the BEST in CLASS practices increased their students’ academic engagement. They described how practices fostered perseverance in their students by making them more willing to attempt and complete challenging tasks. Elaborating on student’s developing work ethic, a teacher noted, “I think it's done wonders for him… using supportive relationships, using that precorrection and really setting him up for success and giving him, I guess, those different opportunities to respond to it have helped him be more willing to work and willing to try things.” Teachers also noticed that their students were more attentive in the classroom because some practices like “opportunities to respond” helped keep them engaged. Referencing “opportunities to respond” and “praise” specifically, another teacher noted, “I think, definitely like, having him respond more, then he's less likely to engage in like off-task behavior and praising him when he does a good job. Like he really needs that a lot…I feel like it helps him a lot.”

Also resulting from practice use, teachers remarked on the changes they witnessed in their students’ willingness to engage in classroom activities. A teacher explained this saying, “So prior to using any of these practices, he was not engaged… he would show up, but he wouldn’t do anything…using supportive relationships…has helped him to be more willing to work and willing to try things.” Teachers also actively fostered academic engagement in focal students by using the BEST in CLASS practices. For instance, a teacher shared that she encouraged her students to continue working hard by making statements like, “oh you made a mistake, so did he. We all did. It’s fine.”

The second theme, *improved academic knowledge, skills, and performance,* comprised two subthemes: improved *academic skills and performance* and *improved students’ understanding and knowledge base*. Approximately 46% of all teachers endorsed this theme. In state A, 25% of teachers shared perspectives alluding to this theme, whereas 56% of teachers in state B expressed similar notions. Their accounts suggested that beyond increases in academic engagement, they also noticed that their students had started performing better academically. One teacher exclaimed that her student’s “math has absolutely skyrocketed.” For other students, their increased academic performance was linked to better engagement and motivation. A teacher shared this saying, “now that he’s engaged in lessons and actually willing to try, he is making some progress.” Relatedly, other teachers saw improvement in some students' understanding of academic concepts and noted this by making statements like “I think that his understanding of the material is definitely something that’s improving.”

The third theme, *improved students’ behavior and the quality of student–teacher relationships*, had three subthemes: *clarified expectations about appropriate behavior, improved students’ general behavior,* and *helped nurture positive student–teacher relationships*. Eighty-five percent of all teachers interviewed endorsed this theme. Within sites, there was also strong endorsement of the themes as 75% of teachers in state A and 89% of teachers in state B expressed views aligned with this theme. BEST in CLASS practices like precorrection helped teachers set clear expectations in their classrooms and helped to ensure that students behaved appropriately. Recounting her use of this practice with a student challenged with writing, one teacher said, “he has a really hard time with writing, just handwriting. So just giving him that precorrection of like, "Here is my expectation. This is what I expect. This is what I want to see from you." And he wants to please me, because we have that relationship now, he tries harder.” This link between precorrection and behavior change was also evident in other students as another teacher explained its impact on her student’s impulsive behaviors by saying, “the precorrection helps his impulses, reminding him what is expected, not the blurting out, and stuff.”

Aside from precorrection, other practices impacted students’ behaviors and resulted in more positive relationships with their teachers. Some teachers reported that their use of the practices helped their students develop informal relationships with them and helped them gain their student’s trust. Describing a friendlier relationship that now existed between herself and her focal student, one teacher mentioned that “that's a lot of why she [her student] is the way that she is with [the teacher]. Because of the praise, because of the supportive relationship. [Her student] knows that she could pick up the phone and she can FaceTime her [the teacher].” This teacher’s comment also highlights specific BEST in CLASS practices like praise and supportive relationships that she believed were associated with the relationship she shared with her student.

The fourth theme, *increased students’ confidence and general comfort,* included accounts about how using BEST in CLASS practices helped students become more comfortable in the classroom and confident in their schoolwork. A smaller proportion of all teachers interviewed (approximately 15%) endorsed this theme, and all statements coded under this theme were shared by teachers in State B. However, these teachers explained that over time, some students became confident about their work. To illustrate, a teacher reported that “whereas in the beginning of the year [she] heard a lot of, I can't do this, now it's, oh, this is easy, I know this…I can do this.”

Similarly, the last theme, no or *unknown effects on academic performance*, was only mentioned by a few teachers (approximately 30%), all from state B, who either saw no improvements in academic performance or were uncertain of their student’s academic progress. Although some teachers believed their students had made progress in certain areas, for students in lower grades, in particular, the lack of graded assignments made it difficult for teachers to make conclusive statements. One teacher explained the challenge by saying, “well, he's definitely made improvements but, um, it's harder in kindergarten to be able to say because I don't, you know, once again, we don't have assignments for grades.” Other teachers were frustrated because, despite their efforts, they were yet to witness gains, particularly in academic performance. For these teachers, being in a remote setting made it even harder to help their students, as another teacher expressed, saying, “I mean (INT: It’s okay), she's not really completing much work. I mean, I'm trying and it's like I can't get over there in her house for her, put her on a computer and make her do it. But I mean, I am trying, and I'm trying with my encouragement and telling her.”

## Discussion

The purpose of this study was to assess the extent to which teachers reported delivering evidence-based practices designed to support elementary school students with or at risk for EBD at least one year after participation in the BEST in CLASS condition of a randomized controlled trial. Additionally, we examined teacher reports on the extent to which they perceived these practices to influence student performance and behavior. We used descriptive phenomenology to explore teachers’ reported use of BEST in CLASS practices as well as their perspective of the practices’ effects on their students’ academic and social/emotional and behavioral outcomes. Importantly, teachers reported on these questions at least one year after having received BEST in CLASS training and practice-based coaching. While all 13 teachers reported using at least one of the BEST in CLASS practices during sustainment, there was variability in reported use. In addition, data indicated five main themes, with teachers reporting that BEST in CLASS practices *(1) increased student’s academic engagement, (2) improved student’s academic knowledge, skills, and performance, (3) improved student’s behavior and quality of student–teacher relationships, (4) improved student’s comfort and general confidence in the classroom,* and *(5) had unknown effects on academic performance.* Below, each of these primary findings will be discussed, followed by a discussion of limitations of the current study and implications for future research.

First, while all teachers reported using BEST in CLASS practices in the sustainment year, there was significant variation in reported use. For example, the majority of teachers reported using rules, supportive relationships, and praise, while less than half of the teachers reported using precorrection and opportunities to respond with their focal students with or at risk for EBD. Of note, precorrection and opportunities to respond are both preventive practices and require some intentionality on the part of the teachers; it is possible that, having received BEST in CLASS practice-based coaching during the implementation year, teachers may have missed the prompting and feedback that trained coaches provided in order to deliver precorrection and opportunities to respond in an intentional way. Additionally, teachers may have been less intentional about their use of practices like opportunities to respond, and therefore, could not actively report on its use and impact. That said, data suggest that teachers were linking practices, which is the final coaching module covered during BEST in CLASS implementation. That is, coaches work with teachers to link BEST in CLASS practices in order to maximize both treatment effects as well as efficiency in delivery in order to promote teacher fluency. Quotes such as “I think it's done wonders for him… using supportive relationships, using that precorrection and really setting him up for success and giving him, I guess, those different opportunities to respond to it have helped him be more willing to work and willing to try things” highlight this teacher’s understanding of the importance of linking these evidence-based practices in order to promote student success.

*Increased academic engagement* was the first main theme identified from the data, and over 75% of the teachers had statements that corresponded with this theme. This theme included subthemes associated with motivation and effort, attention and focus, and engagement and participation in classroom activities. This is important, as these are areas that teachers often struggle with when teaching students with and at risk for EBD (Reinke et al., 2011; Stormont et al., 2005), and teachers reported that the practices helped them actively engage their students. Data from previous studies of BEST in CLASS suggest increases in student engagement (Sutherland et al., 2018), and it is important that teachers connect their practice use with salient student outcomes such as classroom engagement. Again, this is one of the strategies that BEST in CLASS coaches use with teachers; that is, helping them make connections between their practice use and their focal student’s classroom behavior. It is possible that teachers who have received BEST in CLASS practice-based coaching may be more likely in sustainment to still be able to make these connections between their practice use and student outcomes. In addition, while 100% of the teachers at state B schools reported that BEST in CLASS practices were associated with academic engagement, only 1 of 4 teachers at state A schools reported the same; of note, state B schools were mostly in person during the school year, while state A schools were virtual. These data suggest that teachers may have found the use of BEST in CLASS practices most effective during face-to-face instruction, which is not surprising given that this is the modality in which they were trained and coached. In fact, in general across main themes and subthemes, teachers in state B schools reported greater impact of BEST in CLASS practices on student outcomes in the sustainment year than did teachers in state A schools, highlighting the importance of instructional delivery modality in teachers’ perceptions of practice effectiveness.

The second identified main theme, *improved academic knowledge, skills, and performance,* is clearly related to the first theme and was comprised of subthemes focused upon academic skills and performance and students’ understanding and knowledge. Again, over half of the teacher participants from state B reported that BEST in CLASS practices impacted their focal students’ academic knowledge and skills, whereas only one of the four teachers from state A reported the same. This finding may be a function of instructional delivery, with teachers’ perceiving practices to be less effective in a virtual learning context. This is particularly concerning, as those students most vulnerable to negative impacts of the COVID-19 pandemic include students with disabilities and mental health diagnoses (Naff et al., 2022), characteristics associated with students with Tier 2 support needs.

The third main theme, *improved student’s behavior and quality of student–teacher relationships* included corresponding statements from the largest percentage of teachers overall, which is not surprising given that improving student behavior and student–teacher relationships are primary goals of the BEST in CLASS program. Indeed, the subthemes *improved students’ general behavior* and *helped nurture positive student–teacher relationships* included more statements by teacher participants than any other subthemes. Studies have documented improvements in both student and child behavior as well as improvements in teacher–student relationships (Conroy et al., 2022; Sutherland et al., 2018, 2020a, 2020b), and it is encouraging that most teachers continue to note the benefit of BEST in CLASS practices in the sustainment year. However, the final themes and associated teacher statements within *increased confidence and general comfort* and *unclear effects on academic achievement* suggest that some teachers were less able to see the benefits of BEST in CLASS practices, at least in the short term. These findings highlight that for teachers to note more distal effects of BEST in CLASS in these ancillary outcome areas, such as academic performance and student self-confidence, may require additional supports (e.g., sustainment year coaching) or other supplementary intervention practices (e.g., peer tutoring).

### Limitations and Future Directions

While findings from the current study provide some important insights into both how teachers report using BEST in CLASS practices during a sustainment year as well as how effective they perceive these practices to be, several limitations of the current study should be kept in mind while interpreting the results. First, we did not specifically ask teachers whether their use of these practices impacted the specific themes and subthemes identified. Thus, although some teachers did not discuss specific themes in their responses, we cannot assume that they did not hold similar views. This is also important to note given the relatively small number of teachers participating from state A. Future studies probing these specific themes will better elucidate the impact on child outcomes for students in both conditions.

In addition, no quantitative teacher-level data were collected because of COVID-19 related restrictions in our participating schools. Quantitative data would provide a more objective perspective on the impact of practices. Future research could include observational data, when available, to integrate with qualitative data in mixed-method approaches in order to better understand teachers’ sustained use of practices. Also, unlike previous BEST in CLASS research (see Conroy et al., 2022; Sutherland et al., 2018, 2020a, 2020b) we did not systematically screen potential focal students in the current study. Thus, the focal students’ teachers referenced in response to interview questions may have differed from their focal students from the previous intervention implementation year. Related to the findings, or lack thereof, of teachers’ perspectives of practices impacts on students’ academic performance, teacher interviews were conducted relatively early in the school year, making it difficult to objectively assess improvements in academic performance among students in lower grades. The timing of interviews assessing impact of sustained practice use should be considered when examining the impacts of practices on more distal, or ancillary, learning outcomes. Relatedly, having student performance data would be helpful in determining the impact of practices on student outcomes that were not available in the current study.

### Implications for Practice

The findings from this study highlight the value of using evidence-based practices in virtual and in-person contexts on students’ behavioral outcomes. A significant proportion of students exhibit challenging classroom behaviors that can have negative effects on their academic output and mental health later in life (Beyer et al., 2012; Campbell et al., 2006). Therefore, it is encouraging to find preliminary evidence indicating that evidence-based practice use transcends modality to influence behavioral outcomes strongly. This knowledge can facilitate the adoption of hybrid instructional modalities where needed to supplement in-person instruction without increasing the risk of inappropriate behaviors among students.

Nonetheless, our findings also show that although many teachers reported stronger perceived impacts on students’ behavioral outcomes, impressions of impact on academic engagement and performance in the virtual instruction modality were less common. Poor academic outcomes have been linked to increased engagement in challenging behaviors (Katsiyannis et al., 2008), perpetuating a vicious cycle that can place students at risk of greater mental health challenges in the future. Teachers might therefore benefit from receiving more explicit training on the effective use of these evidence-based practices in online settings to increase academic engagement and academic performance.

## Summary

This study examined the extent to which teachers reported delivering evidence-based practices designed to support elementary school students with or at risk for EBD at least one year after participation in the BEST in CLASS condition of a randomized controlled trial. While teachers in the two states who participated in this study were using a mixed delivery of instructional modalities due to the COVID-19 pandemic, findings do suggest that teachers did report using the practices, although some differences were noted across instructional modality and teachers who used a hybrid format were more likely deliver BEST in CLASS practices to their students. In general, most teachers reported that the practices were effective at helping improve student behavior and student–teacher relationships, two targeted outcomes of the BEST in CLASS program. More research is needed to help us better understand the extent to which teachers continue using evidence-based programming once supports, such as training and coaching, and this study is a step in this direction.
